# Critical appraisal of clinical practice guidelines for the management of COVID-19: protocol for a systematic review

**DOI:** 10.1186/s13643-021-01871-7

**Published:** 2021-12-22

**Authors:** Thanansayan Dhivagaran, Umaima Abbas, Fahad Butt, Luckshann Arunasalam, Oswin Chang

**Affiliations:** 1 Faculty of Health Sciences, McMaster University, Hamilton, ON Canada; 2 Faculty of Science, McMaster University, Hamilton, ON Canada

**Keywords:** COVID-19, Systematic review protocol, AGREE II, Critical appraisal, Clinical practice guideline, Guideline assessment

## Abstract

**Background:**

In December 2019, a novel coronavirus, severe acute respiratory syndrome coronavirus 2 was identified as the cause of an acute respiratory disease, coronavirus disease 2019 (COVID-19). Given the lack of validated treatments, there is an urgent need for a high-quality management of COVID-19. Clinical practice guidelines (CPGs) are one tool that healthcare providers may use to enhance patient care. As such, it is necessary that they have access to high-quality evidence-based CPGs upon which they may base decisions regarding the management and use of therapeutic interventions (TI) for COVID-19. The purpose of the proposed study is to assess the quality of CPGs that make management or TI recommendations for COVID-19 using the AGREE II instrument.

**Methods:**

The proposed systematic review will identify CPGs for TI use and/or the management of COVID-19. The MEDLINE, EMBASE, CINAHL, and Web of Science databases, as well as the Guidelines International Network, National Institute for Health and Clinical Excellence, Scottish Intercollegiate Guidelines Network, and the World Health Organization websites, will be searched from December 2019 onwards. The primary outcome of this study is the assessed quality of the CPGs. The quality of eligible CPGs will be assessed using the Appraisal of Guidelines, Research and Evaluation II (AGREE II) instrument. Descriptive statistics will be used to quantify the quality of the CPGs. The secondary outcomes of this study are the types of management and/or TI recommendations made. Inconsistent and duplicate TI and/or management recommendations made between CPGs will be compared across guidelines. To summarize and explain the findings related to the included CPGs, a narrative synthesis will also be provided.

**Discussion:**

The results of this study will be of utmost importance to enhancing clinical decision-making among healthcare providers caring for patients with COVID-19. Moreover, the results of this study will be relevant to guideline developers in the creation of CPGs or improvement of existing ones, researchers who want to identify gaps in knowledge, and policy-makers looking to encourage and endorse the adoption of CPGs into clinical practice. The results of this review will be published in a peer-reviewed journal and presented at conferences.

**Systematic review registration:**

International Prospective Register for Systematic Reviews (PROSPERO)—CRD42020219944

**Supplementary Information:**

The online version contains supplementary material available at 10.1186/s13643-021-01871-7.

## Background

In December 2019, a novel coronavirus, severe acute respiratory syndrome coronavirus 2 was identified as the cause of an acute respiratory disease, coronavirus disease 2019 (COVID-19) [1]. COVID-19 was subsequently declared a pandemic by the World Health Organization on March 11, 2020 [1]. Patients with COVID-19 are typically adults who present with symptoms such as fever, dry cough, myalgia, dyspnea, and fatigue [2]. In contrast, severe cases may present with viral pneumonia, severe acute respiratory distress syndrome, and death [3]. In November 2021, there were over 260 million cases of COVID-19 and more than 5.18 million deaths globally [4]. Given the lack of successful treatments [5], there is an urgent need for a high-quality management of COVID-19.

As a result of the relative novelty of COVID-19, there is a lack of data to help guide prognosis. As such, healthcare providers require high-quality evidence-based tools that can support clinical decision-making and inform medical practice. Clinical practice guidelines (CPGs) are one such tool that healthcare professionals may use to guide clinical practice. CPGs are developed systematically and formulate recommendations based on the highest quality of supporting evidence [6]. They provide healthcare professionals with explicit recommendations on how to proceed and help improve the consistency of care [6]. In response to this pandemic, CPGs have been developed to guide the management of adult patients with COVID-19. High-quality, evidence-based CPGs have the potential to enhance the care of adults with COVID-19; however, this depends on the quality of the CPG and its adoption in clinical practice. Several studies have found that CPGs vary in quality for various health conditions [7–9], and consequently, may affect the healthcare providers’ ability to deliver high-quality care and service. Therefore, in order to enhance care, it is crucial that healthcare providers have access to high-quality evidence-based CPGs upon which they may base decisions regarding the management and use of therapeutic interventions (TIs) for COVID-19.

With an abundance of CPGs available for use by healthcare professionals, a strategy is needed to assess the quality of CPGs, so that those of highest quality are applied in the treatment and management of COVID-19 patients. One well-established and widely used instrument for assessing the rigor and methodological quality of CPGs is known as the Appraisal of Guidelines, Research and Evaluation II (AGREE II) instrument [10]. As a tool for guideline appraisal, the AGREE II instrument seeks to inform the type of information found in CPGs, methodological considerations for guideline developers, and how likely appraisers are to recommend the use of the CPG in professional practice [10]. In particular, the AGREE II instrument is used to assess CPGs across the following six domains: (1) scope and purpose, (2) stakeholder involvement, (3) rigor of development, (4) clarity of presentation, (5) applicability, and (6) editorial independence. By assessing the quality of CPGs across these unique domains, the AGREE II instrument can predict the outcomes associated with the implementation of a CPG, which can greatly inform healthcare practices as they pertain to COVID-19 treatment and management [10].

To date, few studies have examined the quality of CPGs for COVID-19 using the AGREE II instrument [11–15]. These studies found that the recommendations formulated within the CPGs were lacking evidence and that the overall quality of CPGs was poor and variable [11–15]. However, these studies are limited for several specific reasons. Dagens et al. only assessed CPGs for COVID-19 which were published by March 2020, soon after the first confirmed case of COVID-19 [11]; however, the quality of CPGs may have changed since March 2020. Since March 2020, there has been significant growth in COVID-19 research [16], as well as additional time to implement quality improvements in new CPGs for COVID-19. A study by Ong et al. limited its scope to only assessing CPGs on the perioperative anesthetic management of COVID-19 [12]. Similarly, a study by Li et al. limited its scope to only evaluating CPGs related to the treatment of COVID-19 using Chinese herbal medicine [13]. Another study by Yeo et al. limited its scope by population, as it only assessed the quality of CPGs pertinent to the management of COVID-19 in neonates born to mothers with COVID-19 [14]. Due to the limitation in scope, these studies may have obtained results that do not accurately reflect the quality of all CPGs related to the management and/or treatment of COVID-19. Finally, one study by Wang et al. investigated the quality of evidence-based and consensus-based CPGs for the management of COVID-19; however, this study did not differentiate between the quality of CPGs for the management of adults and the quality of CPGs for pediatric populations [15].

To our knowledge, this study will be the first to assess the quality of evidence-based CPGs focused on the treatment and/or management of COVID-19 in adult patients. In doing so, the study will inform healthcare providers on which CPGs should be trusted the most and inform CPG developers of areas that need improvement. Hence, the primary objective of this study is to comprehensively assess the quality of evidence-based CPGs that make management or TI recommendations for adult patients with COVID-19 using the AGREE II instrument. The secondary objective is to determine the types of management and/or TI recommendations made, as well as which recommendations are inconsistent, unique, or duplicate across CPGs.

## Methods

### Study design

The present study will use the AGREE II instrument for the quality assessment of CPGs focused on TI use and/or the management of adult patients with COVID-19 [10]. The present protocol was prospectively registered on the international Prospective Register of Systematic Reviews (PROSPERO) with the following registration number: CRD42020219944. The present protocol has been reported in accordance with the Preferred Reporting Items for Systematic Review and Meta-Analysis Protocols (PRISMA-P) [17] (see PRISMA-P checklist in Additional file 1). The completed systematic review will be reported in accordance with the Preferred Reporting Items for Systematic Reviews and Meta-Analyses (PRISMA) [18]. If significant deviations are made from this protocol, then they will be reported and published with the results of the review.

### Eligibility criteria

The criteria for eligible COVID-19 CPGs were developed in accordance with the Population, Intervention, Comparison and Outcomes (PICO) framework [19]. A CPG is a systematically developed guidance document that makes recommendations for a specific condition based on an analysis of benefits and risks of each intervention [20]. Alternatively, when the evidence for interventions are limited such that an assessment of benefits and harms cannot be accurately made, consensus statements may be developed during a convention of experts [20]. Recommendations are made in a consensus statement if there is sufficient agreement between the expert panel regarding the interventions; however, these recommendations may be biased by those involved in the recommendation development process [20]. Despite these differences between CPGs and consensus statements, both are types of guidance documents [21]. We will include CPGs that focus on adults (18 years or older) with COVID-19. With respect to the interventions, we will include CPGs that primarily discuss and make recommendations for TI use and/or the management of COVID-19. In terms of the TIs that are eligible, we will include all TIs used for the treatment and/or management of COVID-19. These include, but are not limited to, tocilizumab, recombinant angiotensin-converting enzyme 2, remdesivir, lopinavir/ritonavir, anticoagulants, antiviral combination therapies, hydroxychloroquine/chloroquine compounds, colchicine, corticosteroids, oseltamivir/amantadine, and convalescent plasma. We will not limit the inclusion of CPGs to specific TIs, as doing so would prevent a comprehensive assessment of CPGs focused on the management and/or TI use for COVID-19. We will include CPGs that are evidence-based, publicly available, published after 2019, and in English. We will exclude articles that focus on the diagnosis or screening of COVID-19 and the associated SARS-2-CoV-2 virus. COVID-19 diagnosis is commonly done through viral tests such as antigen testing immunoassays and nucleic acid amplification tests [22]. Antigen immunoassays are often employed as a screening tool as they are easy to produce, detect the presence of specific antigen, and indicate current infection [23]. In contrast, nucleic acid amplification tests are viral diagnostic tests that detect the presence of viral genetic material by identifying the ribonucleic acid comprising its genome [23, 24]. CPGs that are not in English or have a more recent update available from each organization will also be excluded. Furthermore, we will also exclude articles that are abstracts, editorials, letters, position papers, protocols, consensus statements, conference proceedings, and summaries of CPGs.

### Outcomes and prioritization

The primary outcome of this study is the assessed quality of eligible CPGs. The assessed quality will be determined through the calculation of the scaled domain percentages for each CPG, as outlined in the AGREE II instrument [10]. We will also report the average appraiser scores and average overall assessments, in addition to the scaled domain percentages. The secondary outcomes of this study are the types of management and/or TI recommendations made. We will determine which recommendations are inconsistent, unique, or duplicate across CPGs.

### Search methods for identification of studies

#### Electronic database search

We will search databases including Medical Literature Analysis and Retrieval System Online (MEDLINE), Excerpta Medica Database (EMBASE), Cumulative Index to Nursing and Allied Health Literature (CINAHL), and Web of Science from December 2019 onwards. As the databases have differing capabilities, we will adapt the search for each accordingly. The searches will use appropriate Medical Subject Headings (MeSH) terms to ensure inclusion of relevant titles and abstracts [25]. We will also search the websites of guideline developing organizations for the most recent guidelines that satisfy the eligibility criteria. A sample search strategy to be run on MEDLINE is presented in Additional file 2. This search strategy may be modified to increase specificity and sensitivity.

#### Other data sources

We will search for relevant documents within CPG organizations, including Guidelines International Network (https://www.g-i-n.net/), National Institute for Health and Clinical Excellence (https://www.nice.org.uk/), Scottish Intercollegiate Guidelines Network (https://www.sign.ac.uk/), and the World Health Organization (https://www.who.int/), as well as grey literature sources.

### Study selection process

Two reviewers will independently perform title and abstract screening using Rayyan [26]. Articles that meet the eligibility criteria will be included and their respective full-texts will be retrieved for full-text screening. Full-text screening will be similarly performed independently and in duplicate. Any discrepancies regarding the eligibility of an article will be resolved through discussion and consensus between reviewers, or recruitment of another co-author for resolution. Supplementary materials and related documents for CPGs that pass full-text screening will be retrieved thereafter. A PRISMA flow chart illustrating the details of the selection process will be provided [18].

### Data collection and extraction

The reviewers will independently and in duplicate perform data collection and extraction in data extraction spreadsheets prepared a priori. Any discrepancies in the data extracted will be resolved through discussion and consensus between reviewers, or recruitment of another co-author for resolution. For eligible CPGs, we will data extract demographic information including the author, title, year of publication, publishing organization, country, and funding source. Any management and/or TI recommendations made will also be extracted in addition to their corresponding level of evidence and grade of recommendation.

### Quality assessment

We will assess eligible CPGs using the AGREE II instrument which is a well-established and widely-validated international tool used to assess the quality and reporting of CPGs [10]. It is composed of 23 items over six quality domains which include the following: (1) scope and purpose, (2) stakeholder involvement, (3) rigor of development, (4) clarity and presentation, (5) applicability, and (6) editorial independence [10]. The instrument uses a seven-point Likert scale that ranges from strongly disagree (1) to strongly agree (7) that each item is met [10]. Each eligible CPG will be evaluated by four appraisers as recommended by the AGREE II instrument [10]. Prior to assessing the CPGs, each reviewer will complete the AGREE II Tutorial + Practice Exercise on the ‘My AGREE PLUS’ platform [27]. The reviewers will also perform a minimum of three rounds of calibration with CPGs that vary in quality. The reviewers will then score the CPGs for each of the 23 items over 6 domains. The overall quality of the CPG (1 to 7) will sequentially be evaluated by the reviewers and used to recommend for or against the use of a CPG. Any major discrepancies in the scoring of a CPG (greater than 3 points) will be resolved through discussion and consensus between reviewers, or recruitment of another co-author for resolution. We will then calculate the average overall assessment scores and average appraisal scores for each CPG. The average overall assessment scores will be calculated by averaging the scores each appraiser gave for ‘overall guideline assessment.’ The average appraisal scores will be calculated by averaging the score an appraiser gave for all 23 items, and then subsequently averaging this value calculated for each of the 4 appraisers. The quality of each domain will be compared within and across CPGs using the scaled domain percentages. The scaled domain percentages will be calculated through the summation of the appraisers’ scores for the items within each domain, and subsequently scaling this value as a percentage of the maximum possible score for the given domain.

### Data synthesis and analysis

Descriptive statistics will be used to quantify the quality of the CPGs. Specifically, we will calculate the average overall assessment scores, average appraisal scores, and the standard deviation for each of these two scores. The scaled domain percentages will also be calculated. We will tabulate these results for ease of quality comparison both across and within the domains of the CPGs. A narrative synthesis will be provided to explain the characteristics and findings of the included CPGs.

### Description and comparison of recommended interventions

We will tabulate the recommendations made in each CPG for the management of COVID-19. Additionally, we will compare inconsistent and duplicate TI and/or management recommendations made between CPGs. Specifically, we will indicate whether a therapy was recommended for or against use in a table. If a therapy was recommended, then it will be indicated with a green box. If a therapy is recommended against, then it will be indicated with a red box. If a therapy recommendation is unclear or conflicting, then it will be indicated with a yellow box. This will allow for ease of comparison between therapy recommendations across the eligible CPGs. In addition to comparing the CPGs in a table, we will also provide a narrative synthesis to describe and compare the recommendations of the included CPGs.

## Discussion

COVID-19 has affected over 260 million people and accounted for more than 5.18 million deaths globally [4]. Classified as a pandemic by the World Health Organization, individuals with COVID-19 suffer from symptoms such as fever, dry cough, myalgia, dyspnea, and fatigue [1, 2], where critical cases may result in severe acute respiratory distress syndrome and death [3]. Many of those individuals affected must turn to healthcare professionals for the high-quality care and management of the condition. Evidently, with the current lack of validated treatments, there is a greater emphasis placed on the management of COVID-19. As such, it is necessary that healthcare professionals have access to high-quality evidence-based CPGs which contain recommendations pertaining to the use of TIs and the management of COVID-19. The purpose of this systematic review will be to investigate the quality of existing CPGs designed for the management of adult patients with COVID-19 using the AGREE II instrument.

### Strengths and limitations

There are several notable strengths to this study, including the use of a comprehensive systematic review to determine eligible CPGs pertaining to TI use and the management of COVID-19, use of multiple appraisers, and the use of the well-established and widely-validated AGREE II instrument.

There also exist some limitations inherent in our proposed study. Firstly, CPGs that are not in English should be excluded; however, this may result in the omission of CPGs pertinent to the proposed research question. Secondly, the target population of the CPGs for this study is all adults over 18 years of age; however, we do not intend on separately analyzing quality between CPGs for adults (18–65 years) from CPGs for elderly adults (65 years and above). It is possible that CPG quality may differ between these target populations. Lastly, given the COVID-19 pandemic, CPG developers may seek to rapidly disseminate knowledge to better manage COVID-19. As a result, CPG developers may prioritize the rapid dissemination of new CPGs instead of prioritizing the methodological quality of CPGs.

### Future implications

Our proposed systematic review and assessment will aide healthcare professionals who wish to improve the standard of care for patients with COVID-19 by incorporating CPGs into their practice, guideline developers in the creation of CPGs or improvement of existing ones, researchers who want to identify gaps in knowledge, and policy-makers looking to encourage and endorse the adoption of CPGs into clinical practice. Additionally, the evidence gathered through this systematic review will provide insight into gaps in available CPGs which may result in the identification of other research avenues.

### Future considerations

Any amendments made to the proposed study will be indicated on PROSPERO and in the “Methods” section of the completed systematic review. This systematic review will be disseminated in a peer-reviewed journal and may be presented at local, national, or international conferences.

## Supplementary Information

**Additional file 1.** Preferred Reporting Items for Systematic Review and Meta-Analysis Protocols 2015 Statement (PRISMA-P checklist): contains items that are required to be reported in a systematic review protocol.

**Additional file 2.** Sample MEDLINE search for COVID-19 Clinical Practice Guidelines executed using the OVID interface: contains sample search strategy on MEDLINE database.

## Data Availability

Not applicable.

## Abbreviations

AGREE II: Appraisal of Guidelines for Research & Evaluation II
COVID-19: Coronavirus Disease 2019
CPG: Clinical practice guideline
PICO: Patients, Intervention, Comparison and Outcomes
PRISMA: Preferred Reporting Items for Systematic Reviews and Meta-Analyses
PRISMA-P: Preferred Reporting Items for Systematic Review and Meta-Analysis Protocols
TI: Therapeutic intervention
