# Supplementary material for: Critical appraisal of clinical practice guidelines for the management of COVID-19: protocol for a systematic review
Source: Syst Rev. 2021 Dec 22;10:317. doi: 10.1186/s13643-021-01871-7 (PMC8694758; doi:10.1186/s13643-021-01871-7)
Supplement: Supplementary file 2 — Additional file 2. Sample MEDLINE search for COVID-19 Clinical Practice Guidelines executed using the OVID interface: contains sample search strategy on MEDLINE database. [file 13643_2021_1871_MOESM2_ESM.docx]

## **Additional File 2: Sample MEDLINE search for COVID-19 Clinical Practice Guidelines executed using the OVID interface**

| # | Searches |
| --- | --- |
| 1 | exp RNA, Viral/ or exp Pneumonia, Viral/ or exp Coronavirus Infections/ or exp Pandemics/ or SARS-CoV-2.mp. or exp Betacoronavirus/ or exp SARS Virus/ |
| 2 | COVID-19.mp. |
| 3 | exp Coronavirus/ or coronavirus.mp. |
| 4 | 2 or 3 |
| 5 | 1 and 4 |
| 6 | exp Practice Guideline/ or guideline.mp. or exp Guideline/ |
| 7 | limit 6 to (english language and yr="2019 -Current") |
| 8 | 5 and 7 |
